# Muscle fiber necroptosis in pathophysiology of idiopathic inflammatory myopathies and its potential as target of novel treatment strategy

**DOI:** 10.3389/fimmu.2023.1191815

**Published:** 2023-07-07

**Authors:** Mari Kamiya, Naoki Kimura, Natsuka Umezawa, Hisanori Hasegawa, Shinsuke Yasuda

**Affiliations:** Department of Rheumatology, Graduate School of Medical and Dental Sciences, Tokyo Medical and Dental University (TMDU), Tokyo, Japan

**Keywords:** necroptosis, inflammatory myopathies, polymyositis, dermatomyositis, HMGB1, reactive oxygen species

## Abstract

Idiopathic inflammatory myopathies (IIMs), which are a group of chronic and diverse inflammatory diseases, are primarily characterized by weakness in the proximal muscles that progressively leads to persistent disability. Current treatments of IIMs depend on nonspecific immunosuppressive agents (including glucocorticoids and immunosuppressants). However, these therapies sometimes fail to regulate muscle inflammation, and some patients suffer from infectious diseases and other adverse effects related to the treatment. Furthermore, even after inflammation has subsided, muscle weakness persists in a significant proportion of the patients. Therefore, the elucidation of pathophysiology of IIMs and development of a better therapeutic strategy that not only alleviates muscle inflammation but also improves muscle weakness without increment of opportunistic infection is awaited. Muscle fiber death, which has been formerly postulated as “necrosis”, is a key histological feature of all subtypes of IIMs, however, its detailed mechanisms and contribution to the pathophysiology remained to be elucidated. Recent studies have revealed that muscle fibers of IIMs undergo necroptosis, a newly recognized form of regulated cell death, and promote muscle inflammation and dysfunction through releasing inflammatory mediators such as damage-associated molecular patterns (DAMPs). The research on murine model of polymyositis, a subtype of IIM, revealed that the inhibition of necroptosis or HMGB1, one of major DAMPs released from muscle fibers undergoing necroptosis, ameliorated muscle inflammation and recovered muscle weakness. Furthermore, not only the necroptosis-associated molecules but also PGAM5, a mitochondrial protein, and reactive oxygen species have been shown to be involved in muscle fiber necroptosis, indicating the multiple target candidates for the treatment of IIMs acting through necroptosis regulation. This article overviews the research on muscle injury mechanisms in IIMs focusing on the contribution of necroptosis in their pathophysiology and discusses the potential treatment strategy targeting muscle fiber necroptosis.

## Introduction

Idiopathic inflammatory myopathies (IIMs) encompass a diverse set of conditions that are characterized by muscle inflammation and progressive weakness primarily affecting proximal muscles. Currently, based on disparities in clinical, histological, and autoantibody features, IIMs are most commonly subclassified into five subsets: polymyositis (PM), dermatomyositis (DM), anti-synthetase syndrome (ASS), immune-mediated necrotizing myopathies (IMNM), and inclusion-body myositis (IBM) (1–6). IIMs are systemic diseases as various organs beyond skeletal muscles are frequently affected. For instance, DM and ASS are accompanied by skin manifestations, and all subtypes of IIMs can be associated with interstitial lung disease, myocarditis, and arthritis. Although the exact pathogenesis of IIMs has yet to be fully clarified, they are presumed to be auto-immune diseases based on the inflammatory infiltrates in muscle tissues with high frequency of T cells, as well as the common presence of autoantibodies including the myositis-specific autoantibodies (MSAs) such as anti-aminoacyl tRNA synthetase (ARS) including anti-Jo-1, anti-transcription intermediary factor 1 γ (TIF1-γ), anti-Mi-2, anti-small ubiquitin-like modifier activating enzyme (SAE), anti-nuclear matrix protein 2 (NXP2), anti-melanoma differentiation associated gene 5 (MDA5), anti-3-hydroxy-3-methylglutaryl coenzyme A reductase (HMGCR), and anti-signal recognition particle (SRP) antibodies (7). Currently, treatments of IIM largely rely on non-specific immunosuppressive agents, such as glucocorticoids and immunosuppressants, including methotrexate, mycophenolate mofetil, azathioprine, and calcineurin inhibitors (3). Glucocorticoids may further deteriorate muscle weakness through glucocorticoid-induced myopathy (8). While some patients suffer from refractory disease despite the immunosuppressive therapies, others experience various adverse effects including infectious diseases. Moreover, muscle weakness remains persistent in over half of IIM patients even after the settlement of muscle inflammation (9). Hence, there is a need to uncover the pathophysiology of IIMs to develop a better therapeutic strategy that improves muscle strength, suppresses inflammation, and mitigates the risk of infection.

Given the limited efficacy of the current treatment strategy targeting immune cells in the resolution of inflammation and recovering muscle strength, a number of researchers have shifted their attention towards the role of muscle fibers in the pathophysiology of IIMs. Muscle fiber death, namely “necrosis”, is a hallmark feature of all subgroups of IIMs (3, 10). Not only do dying muscle fibers contribute to muscle weakness due to the loss of muscle fibers (3), but they also release inflammatory mediators such as cytokines and damage-associated molecular patterns (DAMPs) (11–13), which could potentially promote further muscle inflammation. Several etiologic studies have provided support for this assumption. One study suggested the possible link between intense physical activity and an increased risk of developing IIMs (14). Another study found elevated levels of antibodies against coxsackie B virus in the serum of newly onset cases of juvenile DM (15), implying a potential connection between muscle injury and the development of IIMs. Furthermore, MSAs target intracellular molecules expressed in various cells, including muscle fibers (7). *In vivo* analysis on the mice expressing ovalbumin in their muscles showed that muscle injury led to the proliferation of ovalbumin-specific T lymphocytes (16), suggesting that cell death of muscle fibers could supply intracellular autoantigens and activate immune system (7). Muscle fiber death has been expected to be a novel therapeutic target of IIMs, however, its detailed mechanisms have long remained to be clarified. Recent research including ours has demonstrated that muscle fibers in IIMs undergo a form of cell death called necroptosis (17, 18). Necroptosis is a newly discovered form of regulated cell death that is genetically controlled by specific molecules (19, 20). Considering the pro-inflammatory nature (17, 18, 21) of necroptosis, it holds promise as a potential treatment target for IIMs. This article reviews the muscle fiber injury mechanisms, summarizes research on muscle fiber death, or necroptosis, and its contribution to the pathophysiology of IIMs, and discusses the prospective of necroptosis inhibition as a new treatment strategy.

## Overview of clinical and histological features of DM, ASS, IMNM, and IBM and their proposed muscle injury mechanisms

The muscle injury mechanisms of IIMs have been inferred from the extensive and creditable histological analyses on the muscle specimens of IIM patients. In addition, several animal models of IIMs have been established (22–27) to elucidate the pathophysiology of IIMs. In this section, we provide a concise overview of clinical and histological features, as well as the speculated pathophysiology, of IIM subtypes, except for PM, which will be covered in the next section.

DM is distinct from other IIMs in its characteristic skin lesions, including heliotrope rash, Gottron papules, or Gottron signs (1). DM affects both adults and children, while juvenile DM (JDM) is characterized by systemic vasculopathy and high prevalence of calcinosis (2, 28). Perifascicular atrophy of muscle fibers is a characteristic and diagnostic histopathological finding in DM (4, 29). The upregulation of Major Histocompatibility Complex (MHC) class I in the perifascicular muscle fibers and the deposition of the membrane attack complex, namely C5b-C9, predominantly in the endomysial microvasculature are also distinct in DM, implying the involvement of complement attack and micro vasculopathy in the muscle injury in this subtype (4). It has been postulated that the higher prevalence of CD4^+^ T cells in the endomysium of DM might stimulate B lymphocytes to produce autoantibodies, leading to vascular damage and subsequent muscle fiber death (3, 29, 30). Since the mid-2000s, type I interferon (IFN)-stimulated genes (ISGs), such as MX1, also known as myxovirus resistance A (MxA), have been found to be upregulated in the muscle and skin of clinically diagnosed DM patients, indicating their involvement in DM pathophysiology (31–34). Although the ISG expression has also been observed in the muscles of other subtypes of IIMs (34–36), its level is generally lower than that in DM (35). Among DM cases, higher expression of MX1 is often seen in patients positive for anti-TIF1-γ, anti-Mi-2, anti-SAE, anti-MDA5, or anti-NXP-2 antibodies (34). In contrast, IIM patients positive for anti-ARS antibodies show moderate to low expression of MX1 in muscle fibers (34, 35), with their muscle histopathology characterized by the muscle fiber necrosis and regeneration in the perifascicular region (perifascicular necrosis) (37, 38). The patients exhibit a constellation of clinical features known as ASS, including myositis, interstitial lung disease, arthritis, mechanic’s hand, Raynaud phenomenon, and fever, which distinguishes them from DM (39).

A few murine models have been developed to analyze the role of MSA (26, 27). The Jo-1-mediated myositis model was developed by intramuscular immunization with epitopic peptides of histidyl-tRNA synthetase, also known as Jo-1, along with adjuvant (26). This model demonstrated the CD4^+^ T cell response specific to the antigen and the production of antibodies, whereas mice lacking *Rag2* with impaired antigen-specific T and B cell responses still developed myositis. *Tlr4*-mutant mice showed reduced anti-Jo-1 antibody production but still developed muscle inflammation, suggesting a potential role of innate immunity this model (26). Another model called the TIF1-γ-induced myositis model (TIM) was developed by subcutaneously immunizing mice with recombinant human TIF1-γ with adjuvant (27). TIM demonstrated TIF1-γ-specific T cell response and the production of anti-TIF1-γ antibodies. CD8^+^ T cells played a crucial role in the development of TIM, while CD4^+^ T cells, B cells, and even autoantibodies were less involved. The affected muscle fibers in TIM exhibited upregulation of MX1, and partial inhibition of TIM development was observed in mice deficient in IFN-α/β receptors, indicating the involvement of type I IFNs in this model.

Patients with IMNM typically display progressive proximal muscle weakness and markedly elevated levels of serum creatine kinase (CK) (40–42). Anti-HMGCR or anti-SRP antibodies are often detected in the serum of IMNM patients. Histologically, IMNM is characterized by scattered necrotic muscle fibers with sparse inflammatory infiltrates. Additionally, the deposition of C5b-C9, C1q, HMGCR or SRP, and immunoglobulin G (IgG) on the plasma membrane of non-necrotic muscle fibers (2, 43, 44) is observed. Studies have demonstrated that transferring IgG derived from patients with anti-SRP or anti-HMGCR antibodies into mice results in muscle weakness and muscle fiber necrosis. This effect was attenuated in mice deficient in C3, indicating the involvement of complement activation in the pathophysiology of IMNM (45).

IBM is characterized with progressive and sometimes insidious muscle weakness, particularly affecting distal muscles such as the deep finger flexors or tibial anterior (2, 46). The weakness of knee extensors is more pronounced than that of hip flexors (10, 46), and the weakness can be asymmetrical. Immunosuppressive treatment with glucocorticoids and immunosuppressants is generally ineffective in IBM (3). Histologically, IBM is characterized by the infiltration of CD8^+^ cytotoxic T lymphocytes (CTLs) into the endomysium surrounding and invading non-necrotic muscle fibers (30, 47, 48). These infiltrating CD8^+^ T cells are dominated by CD28^null^ subset, which is considered to be highly cytotoxic (49, 50). IBM is associated with an increase in terminally differentiated KLRG^+^ CD8^+^ T cells in blood and muscles, which are highly cytotoxic and release proinflammatory cytokines such as IFN-γ and TNFα (51, 52). Moreover, MHC class I molecules are upregulated ubiquitously in muscle fibers in affected muscles (4, 53). Clonally expanded CD8^+^ T cells were observed in the peripheral blood and muscles of IBM patients (54), suggesting the involvement of MHC-class I-restricted CTL response. Additionally, characteristic features of IBM muscle histology include congophilic amyloid deposits, autophagic vacuoles, upregulated autophagy-related proteins such as SQSTM1 and MAP1LC3A, and aberrant translocation of TARDBP, an RNA binding protein that serves as splicing regulator, in cytoplasm of muscle fibers (3, 55–57). Recent research showed that aberrant translocation of TARDBP leads to mis-splicing and incorporation of “cryptic” exons. The xenograft model utilizing IBM muscles recapitulated IBM histopathology including the infiltration of CD8^+^ T cells in the endomysium. The effect of T cell depletion on the model was only partially effective, indicating its degenerative nature (58).

## Clinical and histological feature of PM and its pathophysiology; contribution of muscle fiber death

PM is characterized by subacute and progressive proximal muscle weakness (1, 59). Facial, extraocular, and distal muscles are generally not affected in PM (3). Histologically, PM shares similarities with IBM, including the endomysial infiltration of CD8^+^ CTLs (60) that are dominated by the CD28^null^ subset (61–64), as well as the diffuse MHC class I upregulation on the muscle fibers (4, 53, 65). Clonal expansion of CD8^+^ T cells with shared clonotypes in peripheral blood and muscle tissues is also observed in PM patients (66), suggesting a role of CD8^+^ T cells for muscle injury. However, recent unsupervised multivariate analysis, based on autoantibodies and clinical features, indicated that cases initially diagnosed with PM could be reclassified into other subtypes, such as IMNM, ASS, DM, and IBM (5). This suggests that the diagnostic definition for PM is in the process of reorganization, awaiting new classification criteria to be established.

Experimental models have been employed to investigate the pathophysiology of PM. MHC class I mouse model was developed by genetically overexpressing MHC class I K^b^ (H2K^b^) in the muscles of C57B/6 mice (24). It closely mimics the histological feature of PM and is associated with the presence of autoantibodies such as anti-Jo-1 antibodies (24). Studies using this model have shown that H2K^b^ overexpression induces unfolded protein response and endoplasmic reticular stress, resulting in muscle damage and inflammation (24, 67).

C protein-induced myositis (CIM) is another animal model of PM (25). In this model, C57B/6 mice are immunized with recombinant human skeletal C protein fragment, which is a muscle-specific antigen, along with Freund’s complete adjuvant (CFA) (25). CIM mice develop muscle inflammation and a reduction in muscle strength (17, 25, 68). Antibodies against C protein are detected in CIM mice, while MSAs are not (25). Since CIM recapitulates the histological features characteristic of PM, including the infiltration of CD8^+^ CTLs in the endomysium, invasion of CD8^+^ CTLs into non-necrotic muscle fibers, and upregulation of MHC class I in muscle fibers (25), extensive research has been conducted on this model. Autoreactive CD8^+^ CTLs play a crucial role in muscle injury of CIM (25, 69, 70). Furthermore, studies on this model have shown the involvement of innate immunity in muscle tissues in its pathophysiology. Local muscle conditioning with intradermal CFA injection is crucial for inducing myositis in this model, by promoting antigen-presentation for T cells and recruits macrophages that secrete inflammatory cytokines in the muscles (71). While muscle inflammation in CIM spontaneously regresses around 6 weeks after immunization, re-stimulation of local innate immunity with CFA alone can induce a relapse of myositis (71). The “seeds and soil” model of IIMs has been proposed, where autoreactive T cells act as “seeds” and the muscle tissues act as “soil”. This model emphasizes the importance of both the activation of autoreactive T cells and the local conditioning in the development or relapse of IIMs. The importance of local muscle conditioning is also evident in human IIMs, as patients exhibit weakness in limited muscle groups and Magnetic resonance imaging (MRI) often shows a patchy distribution pattern of affected muscles while adjacent muscles remain undamaged (72). Additionally, CD8^+^ CTLs remain in muscles for a prolonged period even after the resolution of the disease (62).

In addition, the research on CIM has provided insights into the role of muscle fiber death in promoting muscle inflammation in IIMs. In CIM, muscle fiber death induced by the intramuscular injection of bupivacaine hydrochloride (BPVC), a local anesthetic, can induce local muscle inflammation and even facilitate the relapse of myositis in mice preimmunized with C protein fragment (13). BPVC injection resulted in the infiltration of CD11b^+^ F4/80^+^ macrophages expressing inflammatory cytokines and chemokines, such as TNFα, IL-1β, and CCL2, into the muscles, indicating that injured muscle fibers can activate local innate immunity, leading to further CD8^+^ CTL-mediated muscle injury in CIM. Moreover, the studies on CIM also revealed the crucial role of IL-23 (73) secreted by macrophages and CD80/86 costimulatory factors (74) in muscle inflammation. These findings suggest the involvement of myeloid cells in connecting the innate immunity with autoreactive T cell activation in the pathophysiology of CIM. Based on these observations, it can be inferred that muscle fiber death plays a role in promoting inflammation in IIMs.

## Necrotic features in muscle fibers of IIMs

Cell death is the process under which a cell ceases to carry out its vital functions culminating in the loss of cellular integrity. Among which, cell death that is strictly regulated by specific molecules is defined as regulated cell death (RCD). In the 1970s, apoptosis was identified as the first type of RCD (75), which is regulated by caspases (76, 77). Upon apoptosis, DNA is cleaved and fragmented by endonuclease, which is activated by cleaved CASP3 (76, 77). Therefore, in addition to the presence of cleaved CASP3, DNA fragmentation, which can be detected using terminal deoxynucleotidyl transferase nick-end labeling (TUNEL) or gel electrophoresis (78, 79), has been widely utilized as a surrogate marker for apoptosis. For many years, apoptosis was considered the only recognized form of RCD, while cell death lacking features of apoptosis was classified as necrosis, an uncontrolled and accidental process of cell death. Apoptosis and necrosis have distinct characteristics: apoptotic cells maintain plasma membrane integrity until completion, displaying molecular signals such as sphingosine-1-phosphate (S1P) and CX3CL1, and phosphatidylserine, which facilitate their swift clearance by phagocytic cells with minimal leakage of intracellular components containing DAMPs and cytokines (80). This makes apoptosis an anti-inflammatory cell death. In contrast, necrosis is characterized by lytic features such as cell swelling, membrane rupture, and absence of DNA fragmentation, resulting in release of intracellular contents including DAMPs and cytokines into the extracellular space, making it a pro-inflammatory form of cell death (76, 81).

Research on muscle fiber death in IIMs was primarily conducted in the 1990s and 2000s. Skeletal muscle fibers have unique structural features, including their large size and multinucleation. Multiple nuclei are believed to contribute to mRNA and protein synthesis required for the contractile machinery in muscle fibers (82, 83). The “myonuclear domain” theory has been proposed, suggesting that each nucleus regulates its transcriptional activity within a specific cytoplasmic volume of the muscle fiber (82, 84). Although this hypothesis continues to be debated (82, 83), this structure is presumed to be beneficial upon injury, as only a limited myonuclear domain undergoes partial cell death, minimizing damage within a large muscle fiber (85). Multiple studies have shown that muscle fibers in IIMs show lytic features without typical characteristics of apoptosis (86–89). This is presumably due to the presence of endogenous anti-apoptotic molecules, such as CFLAR, XIAP, and ARC, and the absence of apoptosis inducing factors, including APAF1 (90–93), in mature muscle fibers. Therefore, muscle fiber death is considered necrosis rather than apoptosis. Muscle fiber death accompanies the leakage of intracellular molecules such as CK, myoglobin, aldolases, and DAMPs, resulting in a pro-inflammatory response. Nevertheless, targeting muscle fiber necrosis has not been a focus in IIM therapy, as necrosis was traditionally viewed as accidental or uncontrollable (76).

Since the 2000s, several types of RCD have been identified within the cell death processes previously classified as necrosis. Such “regulated necrosis” includes necroptosis (19, 20), pyroptosis (94, 95), and ferroptosis (96). While they share common lytic features (76), they can be triggered by various stimuli and executed through distinct signaling pathways. Recent studies have demonstrated the effectiveness of targeting regulated necrosis in models of inflammatory or degenerative diseases (97, 98). Given the pro-inflammatory nature of the cell death in muscle fibers, further elucidation of its mechanism has long been awaited to provide insights into new therapeutic targets for IIMs.

## Mechanisms of muscle fiber death of PM inferred from effector molecules of CTLs

Since research on CIM has highlighted the crucial role of CD8^+^ CTLs in muscle injury in PM (69), investigations into the cell death mechanisms in muscle fibers of PM have focused on the cytotoxic effector molecules of CTLs. PRF1, GZMB, and FASLG are the representative cytotoxic effector molecules of CD8^+^ CTLs with well-described functions in inducing cell death. PRF1 and GZMB, stored in the secretory granules within CD8^+^ CTLs, function cooperatively to induce cell death. Upon recognition of the target cell by a CD8^+^ CTL through T cell receptor (TCR) and formation of an immune synapse, a series of signaling events occur, leading to the reorientation of the microtubule-organizing center (MTOC) within the cell. MTOC guides the secretory granules to the presynaptic membrane. The fusion of these granules with the membrane releases PRF1 and GZMB into the synaptic cleft. PRF1 forms small pores on the postsynaptic cell membrane (99), but the ability of these pores to directly cause cell death is still debated. They appear to be too small to induce cell death under a physiological condition (100, 101). Although they allow the passage of small solutes such as Ca^2+^ and Na^+^, potentially leading to osmotic cell lysis (102), it remains to be determined which specific form of RCD is induced in this context. Meanwhile, for GZMB to exert its cytotoxic effect, it needs to translocate into the cytosol of target cells, which relies on the presence of PRF1 (103). GZMB diffuses across the cell membrane through PRF1-formed pores or enter the cytosol through endocytic uptake coordinated by PRF1 (104–106). Once inside the target cell, GZMB cleaves, or activates, target proteins at specific aspartate residues, resulting in target cell death. GZMB induces cell death via two mechanisms: direct activation of caspases and cleavage of BID, which leads to the activation of mitochondrial cell death pathway (107, 108). Importantly, regardless of the pathway, the cell death induced by GZMB should be apoptosis (76). In the muscles of PM, CD8^+^ CTLs infiltrate the endomysium and express PRF1 and GZMB, which are distributed towards the adjacent muscle fibers (61, 62). In the animal experiments on CIM, the absence of PRF1 attenuated the severity of muscle inflammation and the incidence of myositis (69). However, the suppressive effects of PRF1 deficiency were partial, indicating the involvement of other mechanisms independent of PRF1.

Meanwhile, FASLG induces cell death through binding to its receptor, FAS. Studies in the 1990s showed the expression of both FASLG and FAS on infiltrating lymphocytes and muscle fibers (87, 88) in PM patients. However, since apoptosis was believed to be the only RCD downstream of FAS at that time, and as muscle fibers in PM did not exhibit apoptotic features, FASLG/FAS were not considered to be involved in muscle fiber necrosis (3, 87, 88). Albeit, in the 2000s, regulated necrosis as a type of RCD other than apoptosis was discovered, named as “necroptosis”, which also occurs downstream of death receptors such as FAS, TNFR, and TRAIL (76). Upon FAS stimulation, apoptosis is executed when CASP8 is activated, while necroptosis is induced when CASP8 is absent or inactivated. Several studies revealed that muscle fibers express CFLAR, which functions as an endogenous inhibitor of CASP8 (90–93). Based on these findings, it was speculated that necroptosis could be involved in the cell death in muscle fibers in PM.

## Over-activated necroptosis promotes inflammation

Necroptosis is a form of regulated necrosis that is initiated by the stimulation of death receptors (19, 109), pathogen recognition receptors such as TLRs, and ZBP1 (98, 110). Downstream of death receptors, RIPK1 is phosphorylated and form a complex with FADD and CASP8, leading to the activation of CASP8 (76, 98). Activated CASP8 leads to the activation of CASP3, resulting in apoptosis. Meanwhile, in the absence or inactivation of CASP8, RIPK1 binds with RIPK3 to form a complex known as necrosome (111, 112). The necrosome promotes the autophosphorylation of RIPK3, which then phosphorylates MLKL, leading to its oligomerization and activation (113, 114). Phosphorylated MLKL (pMLKL) translocates to the plasma membrane, where it disrupts membrane integrity and induces necroptosis (115, 116). RIPK1 is therefore crucial in linking death receptor signaling to necroptosis. Additionally, TLRs or ZBP1 can trigger necrosome formation, leading to the phosphorylation of MLKL and necroptosis (117). RIPK1 and RIPK3 are cleaved and inactivated by the proteolytic activity of caspases, including CASP8 (118, 119), therefore, inactivation or absence of CASP8 is necessary for the execution of necroptosis. CFLAR, an inactive homologue of CASP8, binds to CASP8 and inhibits its activation (120), serving as a switch to determine whether the cell undergoes apoptosis or necroptosis. RIPK3 and MLKL are critical for necroptosis execution (76, 98, 113, 114), and their expression or phosphorylation can be utilized as indicators of necroptosis. Specifically, pMLKL is associated with cell membrane disruption and is considered a major surrogate marker of necroptosis (113, 121, 122).

While the physiological relevance of necroptosis is yet to be fully clarified, similar to other forms of cell death, necroptosis is indicated to be involved in the elimination of infected (123) or tumor cells (124, 125), contributing to host defense. However, over-activated necroptosis is regarded as a potent inducer of inflammation. Upon necroptosis, intracellular contents, including DAMPs such as HMGB1, adenosine triphosphate (ATP), histones, and heat shock proteins, as well as IL-33 and other cytokines, are released into extracellular space. These DAMPs can bind to pattern-recognition receptors (PRRs) such as TLR, leading to the activation of various pathways, including NF-κB and NLRP3 inflammasome pathways, especially in myeloid cells (21, 126, 127). In addition, multiple components of necroptosis pathway, including ZBP1, RIPK1, RIPK3, MLKL have been reported to activate NF-κB pathway, promoting the production of inflammatory cytokines (128). RIPK3 also activates NLRP3 and IL-1β-mediated inflammatory responses (129). All these processes contribute to tissue inflammation (17, 18, 21).

Necroptosis has been implicated in a wide range of human diseases (98). Experimental approaches, including genetic ablation and small-molecule inhibitors have shown therapeutic potential targeting necroptosis in animal models of various diseases (98, 130–141). Clinical trials have evaluated the effects of necroptosis inhibitors, including RIPK1 kinase specific inhibitors (GSK2982772, GSK3145095, DLN747, or SAR443122) and Food and Drug Administration (FDA)-approved kinase inhibitors (Pazopanib, Potanib, and Dabrafenib), for inflammatory diseases, neurodegenerative diseases, and cancer (139, 142, 143) (**Table 1**). In the phase 2 study on psoriasis, treatment with GSK2972772 improved the severity of plaque lesion (139). However, clinical trials on GSK2972772 for rheumatoid arthritis (144) and ulcerative colitis (145) failed to show significant improvements in disease activity measures (144, 145). These studies demonstrated good tolerability with minimal side effects (139, 144, 145). As follows, recent research including our own has clarified the involvement of necroptosis in muscle fibers in IIMs and its contribution to their pathophysiology (17, 18).

**Table 1:** Necroptosis inhibitors in clinical trial.

Compound name	Target	Disease condition	Phase	Intervention	ClinicalTrials.gov identifier	Status (March 2023)
GSK2982772	RIPK1	Psoriasis	II	GSK2982772 with placebo	NCT02776033	Completed
		Psoriasis	I	GSK2982772 with placebo	NCT04316585	Completed
		Rheumatoid arthritis	I	GSK2982772 with placebo	NCT02858492	Completed
		Ulcerative colitis	II	GSK2982772 with placebo	NCT02903966	Completed
GSK3145095	RIPK1	Solid tumors	I	GSK3145095 alone or combination with pembrolizumab	NCT03681951	Terminated
DNL747	RIPK1	Alzheimer’s disease	I	DNL747 with placebo	NCT03757325	Completed
		Amyotrophic lateral sclerosis	I	DNL747 with placebo	NCT03757351	Terminated
SAR443122	RIPK1	Cutaneous lupus erythematosus	II	SAR443122 with placebo	NCT04781816	Recruiting
		COVID-19	Ib	SAR443122 with placebo	NCT04469621	Completed
		Ulcerative colitis	II	SAR443122 with placebo	NCT05588843	Recruiting
Pazopanib	RIPK1	Soft tissue sarcoma	I/II	Pazopanib with HDM201	NCT05180695	Recruiting
		Renal cell carcinoma, Soft tissue sarcoma, Metastatic disease	I	Pazopanib with AR-42	NCT02795819	Terminated
		Renal cell carcinoma	II	Pazopanib followed by everolimus	NCT01545817	Terminated
Ponatinib	RIPK1/ RIPK3	Chronic Myeloid Leukemia, Philadelphia chromosome positive acute lymphoblastic leukemia		Expanded access	NCT01592136	Approved for marketing
		Squamous cell lung and head and neck cancers	II/III	Single agent	NCT01761747	Terminated
		Gastrointestinal stromal tumor	II	Single agent	NCT01874665	Completed
Dabrafenib	RIPK3	Melanoma	I/II	Dabrafenib with trametinib with hydroxychloroquine	NCT03754179	Recruiting
		Metastatic Melanoma	I/II	Combination with dabrafenib and trametinib	NCT02392871	Completed
		Erdheim-Chester disease	II	Dabrafenib with trametinib	NCT02281760	Completed

## Muscle fibers in IIMs express necroptosis-associated molecules, and necroptosis inhibition ameliorated *in vivo* and *in vitro* models of PM

Histological examinations of the muscle tissue from PM and DM patients have revealed distinct findings regarding cell death mechanisms. The injured muscle fibers generally lacked typical features of apoptosis (3, 17, 87, 88). Meanwhile, PAX7^+^ satellite cells, also known as muscle precursor cells which contribute to muscle regeneration upon injury (146), showed apoptotic feature such as TUNEL positivity (17). In contrast, the injured muscle fibers in PM patients, who meet the Bohan and Peter criteria (59) and 2017 European League Against Rheumatism/American College of Rheumatology (EULAR/ACR) classification criteria for adult and juvenile idiopathic inflammatory myopathies (1), expressed necroptosis-associated molecules such as RIPK1, RIPK3, MLKL, and pMLKL (17). CASP8 was detected in both PAX7^+^ satellite cells and the injured muscle fibers, but the active subunit of CASP8 was observed only in the satellite cells. In addition, FAS and CFLAR were upregulated in the injured muscle fibers, suggesting that FAS-mediated necroptosis may occur in the muscle fibers in PM. Similar histological findings were observed in the muscle specimens from patients with DM, including those positive for anti-ARS or anti-TIF1γ antibodies (17, 18). In the muscle tissue of the patients with IMNM, elevated expression levels of RIPK3, MLKL, and their phosphorylated forms were observed (18). In the analysis on the patients with DM and IMNM, the mRNA levels of RIPK3 and MLKL in muscle specimens correlated with indicators of myositis disease activity such as serum CK level and manual muscle testing scores (18).

Beyond histological examination, *in vitro* models utilizing C2C12 mouse myoblast cell line have been employed to investigate the mechanisms of RCD in muscle fibers in IIMs (18, 147, 148). One such *in vitro* model focuses on CD8^+^ CTL-induced muscle injury, which is implicated in the pathophysiology of PM (147). This model utilizes a model antigen peptide derived from ovalbumin (OVA) and OT-I CTLs, which recognize OVA presented by H2K^b^. C2C12 myoblasts represents satellite cells and can differentiate into multinucleated myotubes, which represent muscle fibers *in vitro* (149, 150). C2C12 myoblasts or myotubes that are transduced with H2K^b^ and OVA are cocultured with OT-CTLs to analyze the antigen-specific CTL-mediated muscle injury. The analysis using this model demonstrated that the CTL-mediated cell death of myoblasts was dependent on PRF/GZMB and exhibited features of apoptosis. Meanwhile, the cell death of differentiated myotubes was dependent on FASLG. The myotubes co-cultured with OT-I CTLs expressed necroptosis associated molecules including pMLKL. CTL-mediated cell death in myotubes was inhibited by necrostatin-1s (Nec1s), a necroptosis inhibitor that targets RIPK1 kinase (151) or the downregulation of *Ripk3* with small interfering RNA (siRNA) in myotubes, confirming the cell death of myotubes was mediated by necroptosis (17). Furthermore, co-culture of myotubes with OT-I CTL led to elevated levels of HMGB1 and pro-inflammatory cytokines in the culture supernatants. Inhibition of necroptosis with Nec1s in the myotubes suppressed the increase in the levels of these inflammatory mediators, indicating the pro-inflammatory nature of muscle fiber necroptosis (17). Another research group demonstrated that undifferentiated C2C12 myoblasts could undergo necroptosis when stimulated with TNFα and z-VAD-fmk, a pan-caspase inhibitor (18). The cell death was accompanied by elevated expression of MLKL and was inhibited by Nec1s or downregulation of *Mlkl* using single-guide RNA (18, 152). These findings support the occurrence of necroptosis in muscle fibers in IIMs.

The therapeutic potential of necroptosis inhibition in IIMs was assessed using CIM. Injured muscle fibers in CIM expressed necroptosis-associated molecules including RIPK1, RIPK3, MLKL, and pMLKL as well as CFLAR and FAS. Mice lacking *Ripk3* or *Mlkl* showed reduced necrotic muscle area and inflammation compared to wild-type mice (17). Treatment with Nec1s for CIM decreased necrotic muscle area, alleviated muscle inflammation, and improved the grip strength. Levels of pro-inflammatory cytokines in the muscles were also reduced in Nec1s-treated mice (17). However, inhibition of other forms of regulated necrosis, such as ferroptosis and pyroptosis with their inhibitors did not alleviate muscle inflammation or improve grip strength in CIM (17). These findings suggest that necroptosis plays a significant role in the pathophysiology of CIM.

## Contribution of DAMPs in muscle inflammation and muscle weakness

DAMPs released following necroptosis are considered to be involved in pro-inflammatory nature of necroptosis (17, 18, 21). Injured muscle fibers of patients with PM, DM, including those with anti-TIF1γ and anti-ARS antibodies, IBM, and IMNM showed elevated cytoplasmic expression of HMGB1 compared to normal muscle fibers in the same patients (11, 17, 18, 153). The muscle fibers with aberrant HMGB1 expression also highly expressed the necroptosis-associated molecules (17, 18). In addition, in the analysis on muscles of DM and IMNM patients, the muscle fibers with necroptotic features also expressed IL-33, another major DAMPs, which are released subsequent to necroptosis (18). There exist a few reports on the relationship between DAMPs and the clinical feature of IIMs. HMGB1 expression in the muscles of IMNM and IBM patients correlated with disease activity measures of IIMs (153). Serum level of HMGB1 was higher in patients with PM and DM compared to healthy controls (12, 153), and the level was even higher in the patients with interstitial pneumonia (12).

Besides recruitment of inflammatory cells through forming a complex with CXCL12 (154), HMGB1 has innate adjuvant effects on myeloid cells in which it enhances antigen presentation and promotes inflammatory cytokine production through binding to PRRs, such as TLR2, TLR3, TLR4, and TLR9 (155, 156). These TLRs are also upregulated on the muscle fibers of patients with IIMs (12). *In vitro* studies on muscle fibers have demonstrated that HMGB1 upregulated MHC class I expression, enhanced endoplasmic reticulum stress, and impaired Ca^2+^ influx to muscle fibers, resulting in muscle fatigue and dysfunction (12, 157).

**Figure 1:**
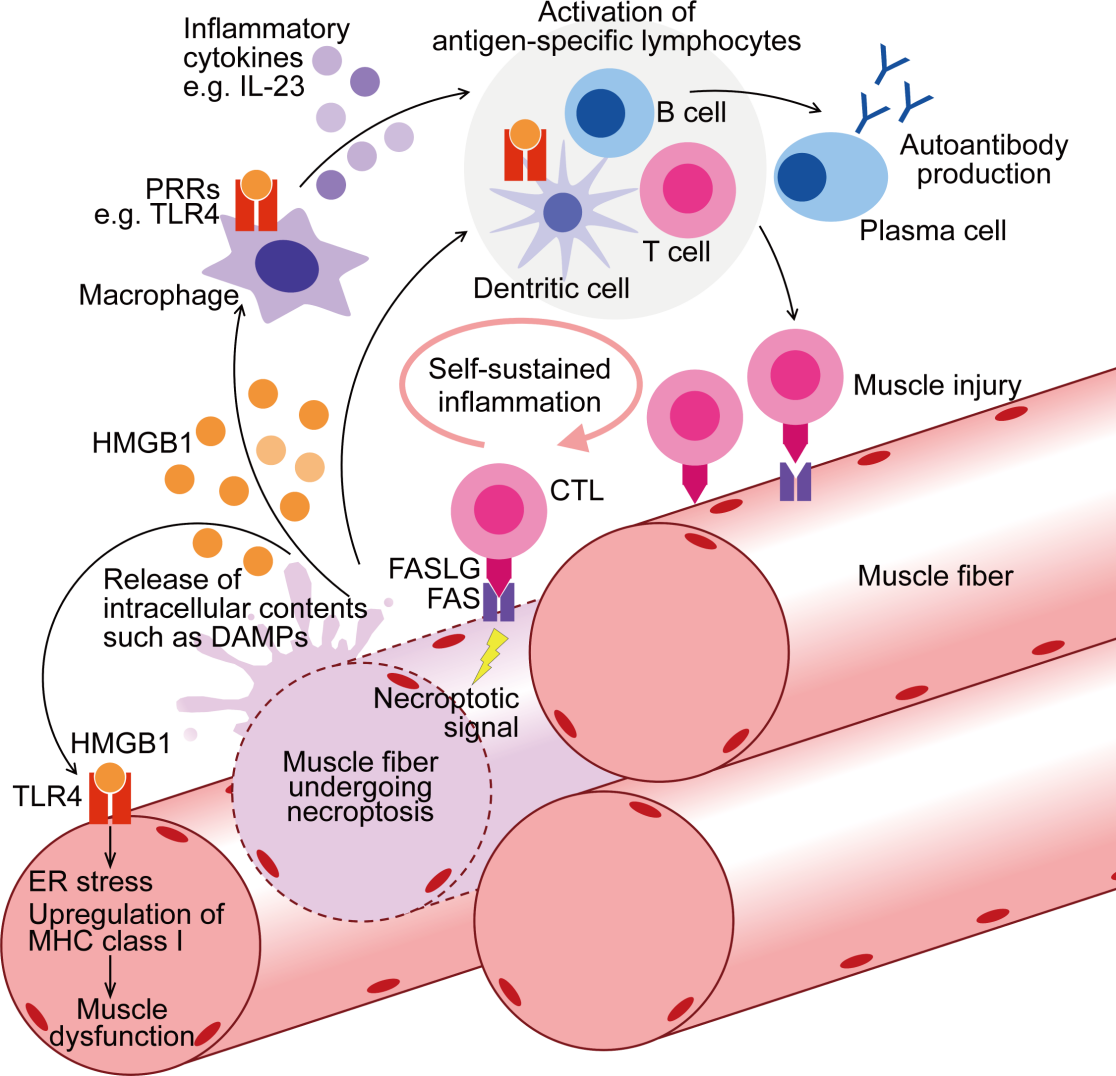
Contribution of muscle fiber necroptosis to pathophysiology of PM. In PM patients, following the injury by CTLs, muscle fibers undergo FAS-mediated necroptosis. During necroptosis, intracellular contents, including DAMPs such as HMGB1, are released to extracellular space. Through binding to PRRs such as TLR4, HMGB1 activates myeloid linage cells including macrophages and dendritic cells, enhancing their antigen presentation and inflammatory cytokine production. These innate inflammatory responses thereby contribute to the activation of the acquired immunity including autoreactive T cell activation and further muscle injury. PRRs are also upregulated in the muscle fibers of PM, and HMGB1 induces ER stress, upregulation of MHC class I, and muscle dysfunction. To summarize, through both inflammatory and non-inflammatory effects, necroptosis in muscle fibers contributes to the pathophysiology of PM. CTL, cytotoxic T cell; DAMPs, damage-associated molecular patterns; PRRs, pattern-recognition receptors; ER, endoplasmic reticulum; MHC, Major Histocompatibility Complex.

HMGB1 upregulation has been confirmed in murine models of IIMs, including the MHC class I mouse model of myositis (24, 67), experimental autoimmune myositis (EAM) (158), and CIM (17). In CIM, serum level of HMGB1 was markedly increased compared to that in control mice, and inhibition of necroptosis with Nec1s decreased its level (17). Antibody-mediated inhibition of HMGB1 (159) on CIM reduced muscle inflammation and retained grip strength (17). Given the potential impact of HMGB1 on both immune cells and muscle fibers (12, 157), HMGB1 could be the novel treatment target for IIMs, addressing both inflammation and muscle weakness (**Figure 1**). However, long-term blockade of HMGB1 in the chronic phase of the disease might impede muscle strength recovery since HMGB1 also plays a role in muscle regeneration through its other receptor, RAGE (160). Nonetheless, the promising therapeutic effects of necroptosis inhibition on the murine PM model suggest that it could be a favorable treatment option, particularly during the acute phase of the disease when muscle fibers are actively damaged by immune cells.

## Potential treatment strategy for IIM targeting muscle fiber necroptosis; glucagon-like peptide-1 receptor agonist

Generally, RCD is closely related with various intracellular factors other than the molecules directly involved in cell death execution. Since the detection of necroptosis in muscle fibers in IIM, the mechanisms or molecules involved in the pathways other than RIPK1, RIPK3, and MLKL have been investigated.

Reactive oxygen species (ROS) have been implicated in various types of RCD, including apoptosis, ferroptosis, necroptosis, and pyroptosis (161–165). An *in vitro* study using C2C12-derived myotubes and recombinant FASLG showed that FASLG-mediated necroptosis of myotubes was accompanied by the intracellular ROS accumulation. Furthermore, ROS promoted myotube necroptosis, implying a role for ROS in the execution of muscle fiber necroptosis (148).

Mitochondrial dynamics, specifically the regulation of mitochondrial volume and function through their fragmentation and fusion, are crucial for maintaining cellular homeostasis. Dysregulation of mitochondrial dynamics can trigger various forms of RCD (166). PGAM5, a protein localized in mitochondria, has a crucial role in mitochondrial fragmentation and serves as a point of convergence for necroptosis in certain cell types (167). In patients with PM and DM, PGAM5 was highly expressed in injured muscle fibers. In an *in vitro* study, FASLG-mediated necroptosis of myotubes was accompanied by upregulation of PGAM5, but was suppressed by downregulation of PGAM5 using siRNA. Consequently, PGAM5 is critical in muscle fiber necroptosis in IIMs (148). Additionally, in myotubes, PGAM5 is regulated by AMP-activated protein kinase (AMPK) (148), which is consistent with other cell types (168).

**Figure 2:**
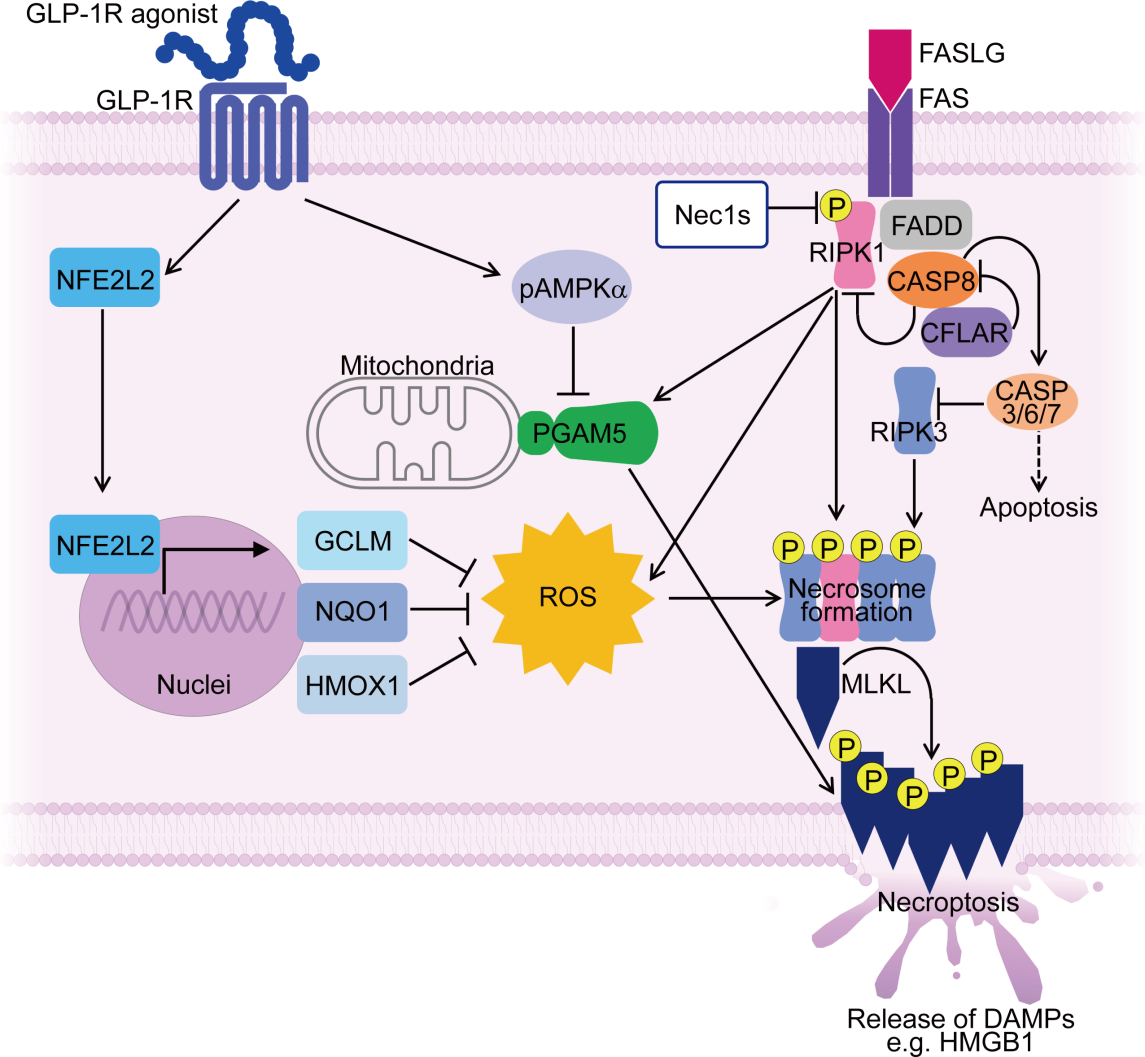
Mechanisms controlling muscle fiber necroptosis in PM and effect of GLP-1R agonist on inhibiting muscle fiber necroptosis. In PMs, PGAM5 and ROS contribute to necroptosis in muscle fibers, along with necroptosis-associated molecules such as RIPI1, RIPK3 and MLKL. PGAM5 is upregulated in necroptotic muscle fibers and serves as a convergence point for necroptosis execution. ROS accumulation during necroptosis further enhances the necroptotic process through promoting necrosome formation. A GLP-1R agonist suppresses the expression of PGAM5 in AMPK dependent manner. Furthermore, the GLP-1R agonist upregulates antioxidant molecules such as NFE2L2, HMOX1, GCLM, and NQO1 in muscle fibers, suppressing ROS accumulation. By targeting PGAM5 and ROS, the GLP-1R agonist inhibits muscle fiber necroptosis, thereby ameliorating PM models. ROS, reactive oxygen species; pAMPKα, phosphorylated AMP-activated protein kinase α; DAMPs, damage-associated molecular patterns; Nec1s, necrostatin-1s. P stands for phosphorylated form.

We have identified a glucagon-like peptide-1 receptor (GLP-1R) agonist as a potential treatment strategy for IIMs targeting muscle fiber necroptosis through regulating ROS and PGAM5. While GLP-1R agonists have been clinically used as an anti-diabetic therapy based on their ability to promote insulin secretion by pancreatic β cells, they possess pleiotropic functions, such as anti-inflammatory effects (169), inhibition of cell death (170), and suppression of muscle atrophy in various disease models (170–173). GLP-1R was upregulated in inflamed muscle fibers of PM and DM patients (148). Treatment with a novel GLP-1R agonist, PF1801, improved CIM-induced muscle weakness, muscle atrophy, and muscle inflammation. PF1801 treatment markedly decreased the level of HMGB1 and other pro-inflammatory cytokines in the serum of CIM. In addition, *in vitro*, PF1801 suppressed FASLG-mediated myotube necroptosis through downregulating PGAM5 in an AMPK-dependent manner. Furthermore, PF1801 upregulated antioxidant molecules such as *Nfe2l2, Hmox1*, *Gclm*, and *Nqo1* and inhibited ROS accumulation of in myotubes (148). Considering the generally low expression level of GLP-1R on immune cells, it has been postulated that the major mechanism through which PF1801 ameliorated the PM model is by inhibiting necroptosis in muscle fibers through the regulation of ROS and PGAM5 (148) (**Figure 2**).

The accumulation of ROS and mitochondrial dysfunction have been observed in both muscle fibers (174–176) and CD14^+^ monocytes (177), in patients with DM/JDM. In muscle fibers, dysregulated mitochondria not only lead to muscle dysfunction through impaired oxidative phosphorylation but also contribute to ROS accumulation and upregulation of ISGs (175–177). In monocytes, defective mitochondrial function can cause ROS accumulation, leading to the production of type I IFNs. ROS can oxidize mitochondrial DNA (mtDNA), yielding dysregulated mitochondria, thereby perpetuating this “ROS” cycle (177). Antioxidant therapy with *n*-acetylcysteine (NAC) suppressed the ISG expression in both muscle fibers (176) and monocytes (177) *in vivo*. NAC treatment in EAM preserved muscle strength, suppressed inflammation, and reduced ISG expression in muscles (176). Additionally, recent study have indicated the involvement of neutrophil extracellular traps (NETs) in the pathology in JDM (178). NETs release requires ROS (178), and NETs containing oxidized mtDNA are highly potent in activating type I IFN signaling (179). While the expression of GLP-1R on immune cells in DM is yet to be clarified, it is speculated that GLP-1R agonists could exert their effects on DM/JDM by targeting ROS, mitochondrial dysregulation, and ISGs.

Conclusively, GLP-1R agonists could be expected as a novel treatment strategy for IIMs through its wide range of actions including inhibition of muscle fiber necroptosis. While GLP-1R agonists have a well-established safety profile, they have dose-dependent side effects such as gastrointestinal dysfunction and weight loss, which may impact treatment adherence and persistence (180). Nevertheless, this strategy could be a viable option for patients, particularly those with comorbid diabetes or obesity.

## Challenges and prospects of treatment strategy for IIMs targeting necroptosis

In summary, emerging studies have shown that injured muscle fibers of IIMs undergo necroptosis. Muscle fibers undergoing necroptosis release inflammatory mediators such as HMGB1 that contribute to muscle inflammation and dysfunction. Targeting necroptosis has shown promising effects in an animal model of IIM, and clinical trials are awaited to evaluate its efficacy and side effects in IIM patients.

The expression of necroptosis-associated molecules in necrotic muscle fibers is a common histological feature shared by different subtypes of IIMs. Meanwhile, the proportion of damaged or necrotic muscle fibers may vary among different subtypes, indicating potential differences in the impact of necroptosis on their pathophysiology. Moreover, many of the molecules or mechanisms believed to play a role in muscle injury in IIM subtypes are not directly linked to known necroptosis machinery, except for FASLG in PM and IFNs (which can induce necroptosis via upregulating ZBP1) in DM. It is also unclear whether necroptosis is the predominant form of RCD in muscle fibers in IIMs. The involvement of other form of cell death such as pyroptosis was implied in DM and EAM model (181, 182). To gain an overall picture of muscle injury and RCD mechanisms in IIMs, comprehensive and dynamic analysis beyond the histological examination is warranted. Currently, the efficacy of necroptosis inhibition in animal model of IIM has only been demonstrated in CIM (17, 148). Given the heterogeneity of IIM subtypes, it would be necessary to develop disease models that recapitulate the clinical and histological characteristics of each IIM subtype, to examine the involvement of cell death utilizing these models. Nonetheless, upregulation of necroptosis-associated molecules, such as RIPK3 and pMLKL, has been observed in injured muscle fibers of patients with Duchenne muscular dystrophy (DMD) and in a mouse model of DMD called mdx mice (152). Genetic ablation of *Ripk3* in mdx mice attenuated muscle fiber degeneration and inflammatory infiltrates and improved muscle function (152). These findings suggest that necroptotic features may be present in diseased muscle fibers, regardless of underlying injury mechanisms. This could be attributed to the super-anti-apoptotic nature of muscle fibers, characterized by distinct expression patterns of anti-apoptotic or apoptosis-associated molecules (90–93). Given the promising results in animal experiments, targeting muscle fiber necroptosis could be effective for a broad spectrum of myopathies, especially those with marked muscle fiber damage or necrosis.

While IIMs are systemic diseases that can affect various extra muscular organs, including skin, lung, heart, joint, and gastro-intestinal tract (65), the impact of necroptosis inhibition on these organs has not been assessed *in vivo*. Nevertheless, since necroptosis has been implicated in inflammatory conditions affecting various organs, and necroptosis inhibitors have shown efficacy in animal models of dermatitis (139, 140), acute respiratory distress syndrome (131, 141), myocarditis (183), arthritis (132), and colitis (184) without apparent side effects, systemic necroptosis inhibition may also be beneficial in treating extra muscular manifestations of IIMs.

Necroptosis functions as an alternative form of RCD that bypasses apoptosis resistance. Many terminally differentiated cells, such as cardiomyocytes, neurons, keratinocytes, and muscle fibers, exhibit resistance to apoptosis. While this resistance is important for tissue maintenance and homeostasis, it can lead to tissue damage through necroptosis under pathologic conditions (136, 140, 183, 185). In addition to the potential role in infectious diseases (123) and tumor immunity (124, 125), necroptosis may contribute to tissue regeneration by releasing inflammatory mediators or regenerating factors. In a murine acute muscle injury model, muscle fiber necroptosis promoted muscle progenitor cell proliferation via releasing TNC, a regenerating factor (186). It is crucial to consider the potential side effects of long-term inhibition of necroptosis for clinical application.

Accordingly, inhibition of necroptosis in muscle fibers holds great promise as a therapeutic strategy to mitigate muscle inflammation and weakness in IIMs. The advantage of this approach is that it targets muscle fibers without directly suppressing immune cells or inflammatory mediators. This could lower the risk of infectious complications and have the potential to restore muscle strength. Further experiments beyond histological analysis are needed to confirm the definitive role of necroptosis in muscle fibers in the pathophysiology of different IIM subtypes. These studies are critical for advancing the clinical application of this discovery and improving treatment options for IIM patient.

## Author contributions

MK, NK, NU, HH, and SY wrote the manuscript. SY supervised the work. All authors contributed to the article and approved the submitted version.
